# Mathematical modeling of laser lipolysis

**DOI:** 10.1186/1475-925X-7-10

**Published:** 2008-02-29

**Authors:** Serge R Mordon, Benjamin Wassmer, Jean Pascal Reynaud, Jaouad Zemmouri

**Affiliations:** 1 INSERM U 703 – IFR 114, Lille University Hospital, 59037 Lille, France; 2 OSYRIS Lasers et Applications 121 Rue Chanzy, 59260 Hellemmes, France; 3 Cemaform, 83000 Toulon, France

## Abstract

**Background and Objectives:**

Liposuction continues to be one of the most popular procedures performed in cosmetic surgery. As the public's demand for body contouring continues, laser lipolysis has been proposed to improve results, minimize risk, optimize patient comfort, and reduce the recovery period. Mathematical modeling of laser lipolysis could provide a better understanding of the laser lipolysis process and could determine the optimal dosage as a function of fat volume to be removed.

**Study design/Materials and Methods:**

An Optical-Thermal-Damage Model was formulated using finite-element modeling software (Femlab 3.1, Comsol Inc). The general model simulated light distribution using the diffusion approximation of the transport theory, temperature rise using the bioheat equation and laser-induced injury using the Arrhenius damage model. Biological tissue was represented by two homogenous regions (dermis and fat layer) with a nonlinear air-tissue boundary condition including free convection.

Video recordings were used to gain a better understanding of the back and forth movement of the cannula during laser lipolysis in order to consider them in our mathematical model. Infrared video recordings were also performed in order to compare the actual surface temperatures to our calculations. The reduction in fat volume was determined as a function of the total applied energy and subsequently compared to clinical data reported in the literature.

**Results:**

In patients, when using cooled tumescent anesthesia, 1064 nm Nd:YAG laser or 980 nm diode laser: (6 W, back and forth motion: 100 mm/s) give similar skin surface temperature (max: 41°C). These measurements are in accordance with those obtained by mathematical modeling performed with a 1 mm cannula inserted inside the hypodermis layer at 0.8 cm below the surface. Similarly, the fat volume reduction observed in patients at 6-month follow up can be determined by mathematical modeling. This fat reduction depends on the applied energy, typically 5 cm^3 ^for 3000 J. At last, skin retraction was observed in patients at 6-month follow up. This observation can be easily explained by mathematical modeling showing that the temperature increase inside the lower dermis is sufficient (48–50°C) to induce skin tightening

**Discussion and Conclusion:**

Laser lipolysis can be described by a theoretical model. Fat volume reduction observed in patients is in accordance with model calculations. Due to heat diffusion, temperature elevation is also produced inside the lower reticular dermis. This interesting observation can explain remodeling of the collagenous tissue, with clinically evident skin tightening.

In conclusion, while the heat generated by interstitial laser irradiation provides stimulate lipolysis of the fat cells, the collagen and elastin are also stimulated resulting in a tightening in the skin. This mathematical model should serve as a useful tool to simulate and better understand the mechanism of action of the laser lipolysis

## Introduction

The subcutaneous fat layer, or hypodermis, bridges between the overlying dermis and the underlying body constituents. In most areas of the body this layer is relatively thick, typically in the order of several millimetres. However, there are areas of the body where pockets of excess fat can be observed: abdomen, hips, buttocks, thighs, knees, upper arms, chin, cheeks and neck. Liposuction is a procedure that can help sculpt the body by removing unwanted fat from these specific areas. Liposuction has become increasingly popular over the last decade and is now among the most popular body sculpting procedures. This increasing popularity is associated with the evolution of techniques and equipment for fat removal and body reshaping. Besides the traditional suction-assisted lipoplasty, other options include ultrasound-assisted and external ultrasound-assisted liposuction, power-assisted liposuction, and laser lipolysis. The efforts in the search for alternatives and new tools aim mainly at reducing downtime, decreasing operator effort for the surgeon and assistant, reducing bleeding and promoting skin tightening,

Laser lipolysis, also called laser lipoplasty has been has been described first in 1994 [1]. This technique is now widely used in Europe and Latin America, and has recently been introduced in Japan and the United States. Less trauma, bleeding and pain have been the main advantages of this technique [2].

After adequate infiltration of an anesthetic solution, a flexible fiber optic delivered through a small caliber cannula is inserted inside the fat tissue. The positioning of the 1 mm cannula is highlighted via trans-illumination from a red guiding beam. The laser energy is transmitted to the adipocytes, which absorb the energy, expand their volume, and rupture [3]. Histologic analyses of the effects of the Nd:YAG laser and of the continuous wave 980 nm laser diode on human fat tissue have shown areas of reversible cellular damage (tumefaction), irreversible tissue damage (lysis) and a reduced intensity of bleeding, as compared with the tissue products by conventional liposuction [4,5].

The mechanisms leading to laser lipolysis are temperature dependent. First, for low energy and consequently low temperature, only tumefaction of the adipocytes is observed [4]. Using higher energy, the histological assessment carried out by Goldman on tissues removed immediately following the procedure showed the rupture of adipocytes but also the coagulation of small vessels in the fatty tissue. Since the heat is confined inside the adipocyte, it leads to the rupture of its membrane. The effect is not only thermal but also thermomechanical.

More importantly, the degree of tumefaction and lysis varied proportionally with the intensity of energy accumulated to the target. Badin et al showed that the conventional liposuction produces less reversible damage (tumefaction) than laser lipolysis with 1,000 J of energy [4]. Using energy ranging from 1,000 J up to 12,000 J, Kim et al observed that the higher the energy, the greater the volume reduction. Typically, a 5 cm^3 ^reduction of fat volume is observed with 3,000 J.A 20 cm^3 ^volume reduction is obtained with 12,000 J. All these studies show clearly that two parameters must be considered for laser lipolysis: 1) the wavelength since the interaction of the laser with the tissue is achieved by the absorption of the laser energy by the receptive chromophores, thus producing sufficient heat to cause the desired thermal damage. The heat acts of the fatty cell, the extracellular matrix to produce both reversible and irreversible cellular damage, which facilitates the liposuction through less trauma and bleeding, 2) the energy since it exists a dose-response relationship [6,7].

The aim of this paper is to present a mathematical model of laser lipolysis using dynamic tissue changes based upon the Arrhenius damage model. Numerical simulations are compared to data recorded during a clinical procedure. Fat volume reduction is determined and compared to data previously reported in the literature. Theoretical understanding of laser lipolysis could help to improve the technique and optimal parameters emerging from these calculations can be taken in account to determine optimal laser dosage.

## Materials and Methods

### Mathematical modeling

An opto-thermal model of laser lipolysis consists of calculations of light distribution, temperature rise and the extent of thermal damage. The following sections describe the manner in which each stage has been implemented in our calculations.

### Geometrical description of the model

**Figure 1:**
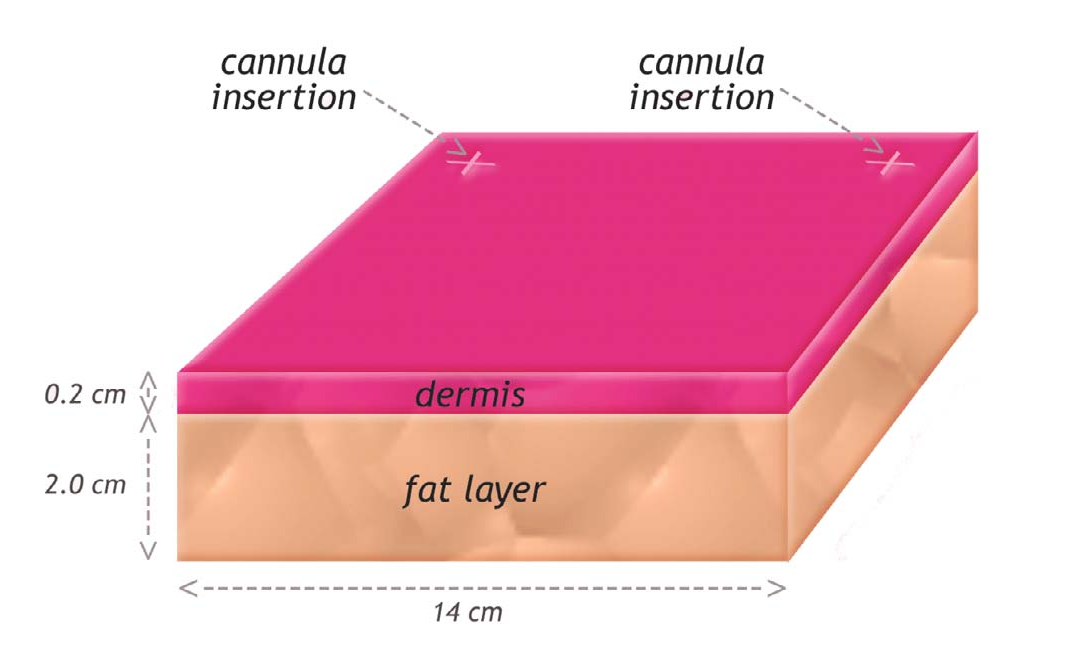
Model geometry: 3D model consisting of a volume of tissue with two different layers: a dermal layer (thickness: 2 mm) and a hypodermis layer (thickness: 20 mm). The dimensions of this volume were 14 cm × 14 cm × 2.2 cm. This volume is s surrounded by infinite and homogenous tissue

The geometry used to simulate was based on a 3D model consisting of a volume of tissue with two different layers: a dermal layer (thickness: 2 mm) and a hypodermis layer (thickness: 20 mm). The dimensions of this volume were 14 cm × 14 cm × 2.2 cm. This volume was surrounded by infinite and homogenous tissue (Figure 1). Calculations were performed for different positions of the laser cannula containing fiber optic inside this volume.

**Figure 2:**
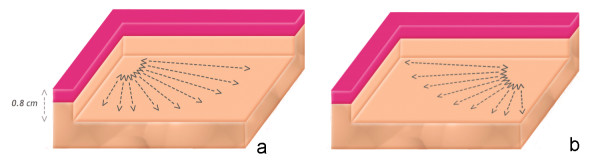
**a**: Model geometry: The cannula is inserted inside the hypodermis layer at approximately 0.8 cm below the surface. This cannula is moved back and forth onto 100 mm in the fat layer with a velocity of 100 mm/in a plane parallel to the surface. This back and forth motion is repeated 15 times for each position of the cannula. This step is repeated 9 times (10 degrees angle each time). **2b**: in order to provide a homogeneous treatment of the fat layer, the cannula is inserted at a distance of 120 mm from the previous insertion point and the procedure is repeated.

The laser lipolysis model used was based on the technique performed by the surgeon during submental laser lipolysis (Figures 2a &2b). Four different steps were identified: #1 – the cannula is inserted inside the hypodermis layer at approximately 0.8 cm below the surface, #2 – this cannula is moved back and forth onto 100 mm in the fat layer with a velocity of 100 mm/s in a plane parallel to the surface. This back and forth motion is repeated 15 times for each position of the cannula. #3 – the angle of the cannula is oriented in the same plane but its angle is modified to cover an angle of 90 degrees. This step is repeated 9 times (10 degrees angle each time). #4 – in order to provide an homogeneous treatment of the fat layer, the cannula is inserted at a distance of 120 mm from the previous insertion point and steps #2, and #3 are repeated a second time.

### Cannula position

The displacement d of the canula in each angular position is described by the following equation:

$$d=a\times \left|\sin^{-1}\left(\cos\left(\frac{\pi .t}{T}-\pi \right)\right)\right|$$

Where:

a (mm): total amplitude of the displacement (100 mm)

T (s): period of each back and forth motion (2 s)

So y_inc _and x_inc _is calculated at each time step with the following equation:

$$\begin{array}{c} x_{inc}=d\times \sin\left(\theta +\frac{\pi }{2}\right)\\ y_{inc}=d\times \cos\left(\theta +\frac{\pi }{2}\right) \end{array}$$

Where:

*θ *(°): angular position incremented of 10° each 30 s.

In order to obtain reproducible results, the surgeon was asked to follow the four different steps described into the protocol. Thanks to conventional video imaging and infrared video imaging, it was possible to ensure that the different steps were effectively performed properly.

### Light distribution in tissue

The light emitted from the fiber inserted the fat layer was modeled as an isotropically radiating point source. As previously proposed by Lizuka et al, spatial distribution has been considered to be dominated by scattering processes [8]. The light irradiance rate (W.mm^-2^) of an isotropic point source emitting P_laser _(W) within an infinite homogeneous medium can be expressed as

$$\varphi (r,t)=\frac{P_{Laser}\exp(-{µ}_{eff}r)}{4\pi Dr}$$

where: *P*_*Laser *_(W): power of the light source

*μ*_*eff *_(mm^-1^): effective attenuation coefficient

*r *(mm): radial distance from the source

*D *(mm): optical diffusion distance

*μ*_*eff *_is determined by the following equation

$${µ}_{eff}=\sqrt{3{µ}_{a}({µ}_{a}+{µ}_{s}^{'})}$$

where *μ*_*a *_(mm^-1^): absorption coefficient in tissue

*μ'*_*s *_(mm^-1^): reduced scattering coefficient: ${µ}_{s}^{'}={µ}_{s}(1-g)$

*μ*_*s *_(mm^-1^): scattering coefficient

*g*: anisotropy factor incorporating the effects of directionally dependent scattering.

*D *(*mm*) is determined by the following equation:

$$D=\frac{1}{3({µ}_{a}+{µ}_{s}^{'})}=\frac{{µ}_{a}}{{µ}_{eff}^{2}}$$

*r *is defined by the following equation

$$r=\sqrt{\left(x²+y²+z²\right)}$$

Where: x, y (mm): transverse dimensions

z (mm): depth

The absorbed power density (W.mm^-3^) is expressed as follows (Welch 1984):

*P*_*abs *_= *μ*_*a*_*φ *(*r*)

The laser irradiation always begins at coordinates (0,0) in Figure 1. The relative position of the cannula inside the fat layer is given by:

*x*' = *x *- *x*_*inc *_and *y*' = *y *- *y*_*inc*_

Where: z_inc _(mm) is the absolute position of the cannula inside the fat layer. The position of cannula is calculated for by taking into account the velocity (mm/s) during the back and forth motion. The relative position of the cannula is obtained by taking into account the cannula velocity:

*x*_*inc *_= *t *× *v *and *y*_*inc *_= *t *× *v*

where: v (mm.s^-1^) is the velocity of the back and forth motion of the cannula inside the fat layer.

### Calculation of temperature rise

Absorption of light in tissue causes a local elevation in temperature. Tissue heat transfer due to the deposited light is described by the following bioheat transfer equation

$$\nabla \cdot k\cdot \nabla T(r,t)+P_{abs}(r,t)-\omega_{b}C_{p}\cdot \left[T(r,t)-T_{art}\right]=C_{p}\frac{\partial T(r,t)}{\partial t}$$

Where

*T *(*r*, *t*): temperature (°K)

*ρ*: density of tissue (g mm^-3^)

*C*: specific heat of tissue (J.g^-1^.°K^-1^)

*C*_*p *_= *C*·*ρ*: *heat capacity *(J.mm^-3^.°K^-1^)

*k *= thermal conductivity of tissue (W. mm^-1^.°K^-1^)

*r *= radial distance (mm)

*t *= time (s)

Values used for calculation are reported in Table 1

**Table 1:** listing of physical parameters used for numerical simulation

		**Hypodermis**	**Dermis**
**Optical coefficients **[17-20]	*μ*_*a *_(mm^-1^)	**0.1**	**0.04**
	*μ*_*s *_(mm^-1^)	**9**	**17**
	*μ'*_*s *_(mm^-1^)	**0.81**	**1.53**
	*g native*	**0.91**	**0.91**
	*μ*_*eff *_(mm^-1^)	**0.52**	**0.43**
	D (mm)	**0.36**	**0.21**
**Thermal coefficients **[21-23]	*C *(J.g^-1^.K^-1^)	**2.87**	**3.3**
	*ρ *(g.mm^-3^)	**0.86.10**^-3^	**1.2.10**^-3^
	*k *(W.mm^-1^.K^-1^)	**3.02.10**^-4^	**4.4.10**^-4^
	wb (ml.100 g^-1^.min^-1^)	**21**	**-**
	wb (g.mm^-3^.s^-1^)	**3.5.10**^-6^	**-**
	h (W.m^-2^.K^-1^)	**-**	**15**
**Tissue Damage Coefficients **[11]			
	E_a _(J.mol^-1^)	**6.28.10**^5^	**6.28.10**^5^
	A (s^-1^)	**3.1.10**^98^	**3.1.10**^98^

- *μ*_*a *_(mm^-1^): absorption coefficient in tissue,

- *μ*_*s *_(mm^-1^): scattering coefficient

- *μ'*_*s *_(mm^-1^): reduced scattering coefficient: ${µ}_{s}^{'}={µ}_{s}(1-g)$

- *g*: anisotropy factor *μ*_*eff *_(mm^-1^):

- *μ*_*eff *_effective attenuation coefficient,

- D (mm): optical diffusion distance

- *C*: specific heat of tissue (J.g^-1^.°K^-1^)

- *ρ*: density of tissue (g mm^-3^)

- *k*: thermal conductivity of tissue (W. mm^-1^.°K^-1^)

- wb: blood flow rate (ml.100 g^-1^.min^-1^)

- h heat-transfer coefficient (W.m^-2^.K^-1^)

- *E*_*a *_(J.mole^-1^): activation energy

- *A *(s^-1^): frequency factor

Convection of the skin surface was calculated using the following equation

∅_Conv _= hSΔT

∅_Conv_: heat flux through the surface (W)

h: convection coefficient (W.m^-2^.°K^-1^)

S: Surface of the interface (m^2^)

ΔT: difference between the inner and the outer temperature (°K)

Boundary conditions for the other surfaces were

$$\vec{n}.k\nabla T=0$$

$\vec{n}$: direction of the heat flux

k: thermal conductivity

### Damage function

Thermal damage in cells and tissue can be described mathematically by a first-order thermal-chemical rate equation, in which temperature history determines damage. Damage is considered to be a unimolecular process, where native molecules transform into a denatured/coagulated state through an activated state leading to cell death. Damage is quantified using a single parameter Ω, which ranges on the entire positive real axis and is calculated from the Arrhenius law [9]. Damage Ω is dimensionless, exponentially dependent on temperature, and linearly dependent on time of exposure.

$$\log(\Omega )=\log(A)+\log(\int_{0}^{\infty }\exp(\frac{-Ea}{RT(r,t)})dt)$$

Where *A *(s^-1^) is the frequency factor,

*E*_*a *_(J.mole^-1^) is the activation energy,

*R *(J.mole^-1^.°K^-1^) is the universal gas constant,

*T *(°K) is the temperature.

Damage Ω is a parameter that is reflective of the extent of damage. A is a frequency factor that describes how often a change in configuration actually occurs when such a reaction is energetically possible, which is also very dependent on molecular structure.

The equation indicates that the measure of damage describes the probability of tissue being destroyed. It is the logarithm of the ratio of the initial concentration of undamaged tissue to the concentration once damage has accumulated, for the time interval t = 0 to t = *τ*. Therefore, Ω = 1 corresponds to an irreversible damage of 100% of the affected cells.

Because the lipid bilayer components of the cell membranes are held together only by forces of hydratation, the lipid bilayer is the most vulnerable to heat damage. Even at temperatures of only 6°C above normal (i.e. 43°C), the structural integrity of the lipid bilayer is lost [10].

In accordance to previous studies, an activation energy equals to 628 KJ/mol and a frequency factor A equals to 3.1e^98 ^s^-1 ^were used in our numerical model for cell membrane lysis [11]

### Numerical implementation

The numerical simulation model was constructed with COMSOL Mutiphysics (COMSOL, Grenoble, France). Using this software, light distribution, bioheat transfer and damage function were solved simultaneously using the finite element method.

An irregular 10 × 60 finite element grid was used. The time steps were 0.1 s. The tolerances used to converge the solution were 10^-3^. The initial temperature was set at 30.4°C since injection of cooled tumescent anesthetics inside the treated volume was performed.

For numerical simulations, parameters commonly used for laser lipolysis were used. Two different wavelengths: 980 nm and 1064 nm were evaluated (power: 6 W), a 400 *μ*m laser fiber introduced in a 1 mm cannula was considered for calculation. The listing of physical parameters used for numerical simulation is reported in Table 1.

Thanks to the numerical simulation, the surface temperature during the laser lipolysis treatment was determined for the different set of parameters and was compared to the surface temperature measured during a clinical procedure with an infrared camera. Several studies have suggested that that the total energy applied in a volume of tissue is the major determinant of treatment outcome in terms of percent reduction of fat volume [6]. In order to compare the results obtained through mathematical modeling, the total energy versus fat volume reduction was calculated.

### Laser procedure

Submental laser lipolysis was performed in one patient in order: 1) to obtain a better understanding of the procedure, in particular on the position and movement of the laser cannula during the procedure, 2) to perform surface temperature measurements during the procedure, 3) to compare two different lasers used for laser lipolysis: a 1064 nm Nd:YAG laser (Smartlipo, Deka, Calenzano, Italy) and a 980 nm diode laser (Pharaon, Osyris, Hellemmes, France).

After obtention of the authorization of the ethical committee, and the informed consent of the patient, laserlipolysis was performed by one of the author (JPR). During this procedure, a mean power of 6W was used for both lasers. The 400 *μ*m laser fiber was inserted inside a 1 mm cannula (Unimed, Lausanne, Switzerland) The procedure was performed after injection of tumescent anesthesia. Duplex control (Aloka 3500, Decines, France) was used to guide injection of 7–8 mL aliquots of the following solution: 10 ml lidocaine 1% with epinephrine and 10 ml lidocaine 1% without epinephrine and additional 60 ml physiologic serum. The injections were performed into the facial space.

### Video recording

Video recording of a submental laser lipolysis was performed using a color video camera 500 × 582 pixels (Model 802P, Radiospares, Beauvais, France). Video recordings were analyzed off line in order to know: 1) the position of the insertion point, 2) the different positions of the cannula during the procedure, 3) the velocity of back and forth movement of the cannula. These data were used for mathematical modeling.

### Infrared video imaging

Infrared video imaging was used to measure the skin surface temperature during laser lipolysis. The skin surface temperature distribution was compared to mathematical modeling of skin temperature elevation following laser lipolysis [12]. An infrared camera (ThermaCAM™ A20, Flir systems™, Issy-Les-Moulineaux, France), mounted with a macro lens giving a 34° × 25° view angle, was used. This camera detected temperature differences as small as 0.10°C in a range from -20°C up to +900°C and produced high-resolution images (160 × 120 pixels). The camera was positioned at a distance of 30 cm from the skin surface allowing infrared imaging of a 20 cm × 20 cm area. The recorded surface temperature were compared to those determined by numerical simulation using similar set of laser parameters.

### Quantification of volume

In order to assess the reduction of volume of the submentum after laser lipolysis, volume reduction was determined using a technique similar to the technique described by Lowe et al. [13]

## Results

### Video recordings

Movie01 shows a laser lipolysis procedure of the submental area [see Additional file 1]. Under cooled tumescent anesthesia, a 1 mm cannula was inserted inside the hypodermis layer at approximately 0.8 cm below the surface (step #1). The surgeon was able to see where the end of the cannula is at all times due to the visible red light of the aiming beam that shined through the skin. This cannula was moved back and forth onto 100 mm in the fat layer with a velocity of 100 mm/s in a plane parallel to the surface. This back and forth motion was repeated 15 times (step #2). Then, the surgeon oriented of the cannula in the same plane but with a different angle (+ 10 degrees). This back and forth motion was repeated again 15 times. This step was repeated 9 times (10 degrees angle each time) to cover the entire area (step #3). In order to provide a homogeneous treatment of the fat layer, the cannula was removed and was inserted again at a distance of 120 mm from the previous insertion point and steps #2, and #3 were repeated a second time.

### Infrared video recordings: surface temperature

**Figure 3:**
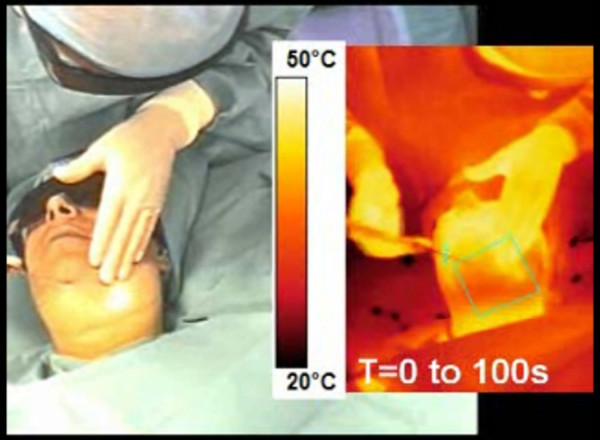
video recording of a laser lipolysis procedure inside the submentum. Conventional and infrared video recordings are performed simultaneously.

Movie01 shows in a different window the infrared video recording of the laser lipolysis of the submental area [see Additional file 1]. The influence of cooled tumescent anesthesia can be clearly seen at the beginning of the procedure. Surface skin temperature was reduced down to 30°C (Figure 3).

**Figure 4:**
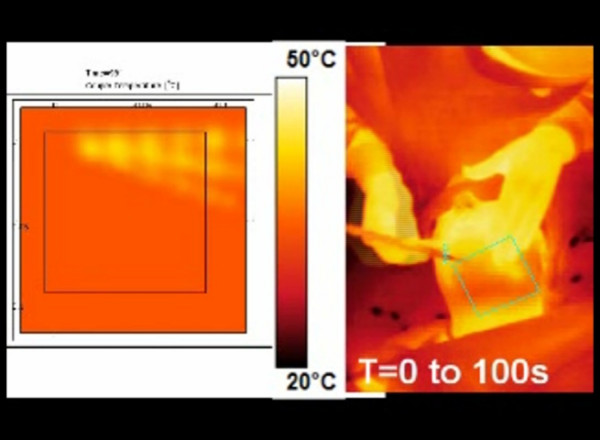
infrared video recording of a laser lipolysis procedure inside the submentum (right) and mathematical modeling performed of a specific area (green square of submentum of the patient).

Movie02 shows the infrared video recording of the surface temperature during 980 nm-laser [see Additional file 2]. In another window, numerical simulation performed with the same laser parameters (980 nm diode laser, power: 6W, CW, back and forth motion: 100 mm/s) is displayed (Figure 4). Mathematical modeling of surface temperature appears to be similar to surface temperature recorded during laser lipolysis of the submentum.

**Figure 5:**
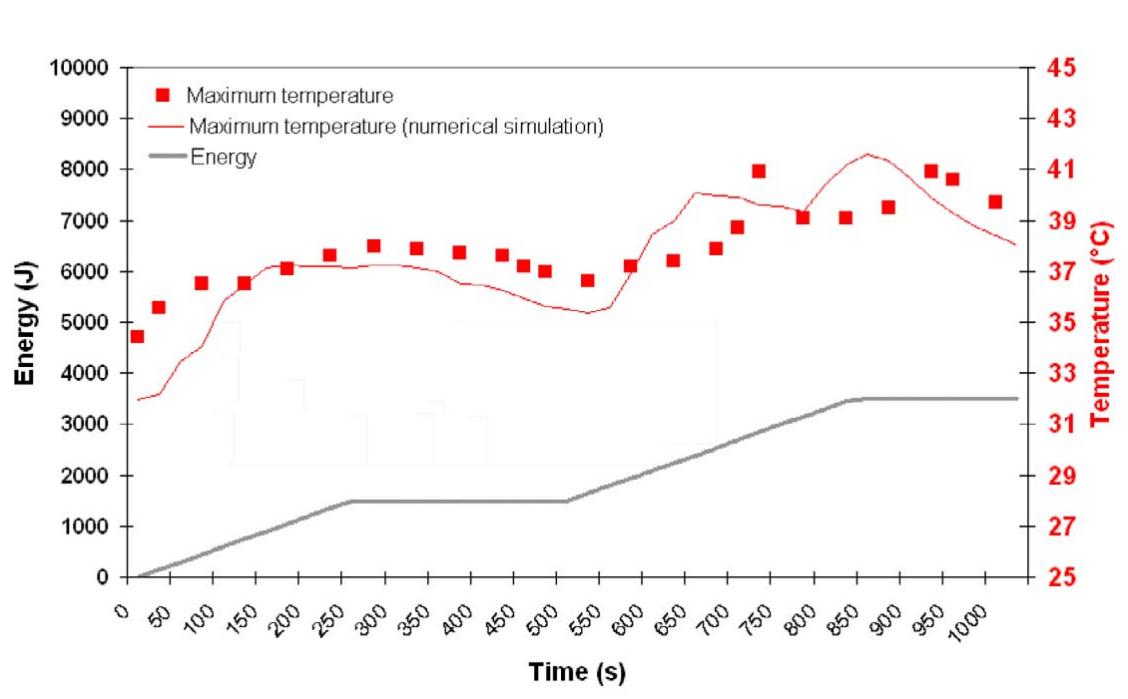
Maximum temperature recorded on skin surface (red square) using the infrared camera and determined at the same position by numerical simulation (red line) as a function time and energy. The energy delivery as function of time is traced (gray line). Right side: 980 nm diode laser, power: 6W, CW, back and forth motion: 100 mm/s).

**Figure 6:**
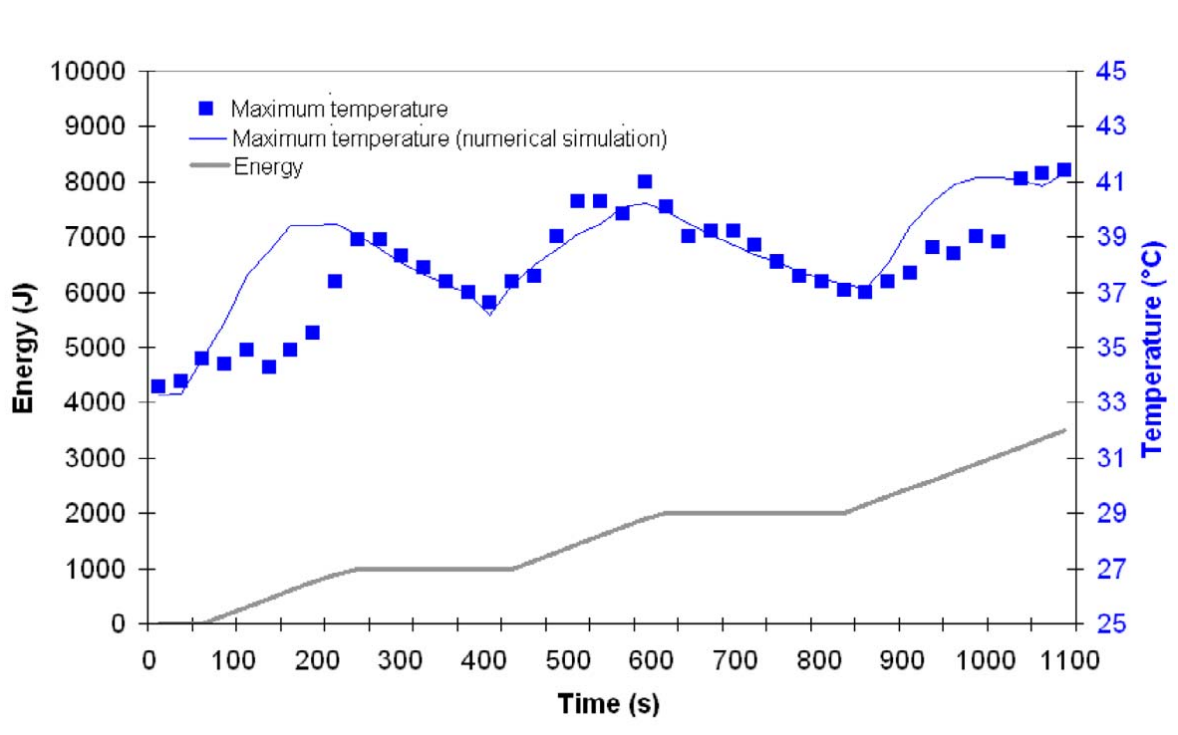
Maximum temperature recorded on skin surface (blue square) using the infrared camera and determined at the same position by numerical simulation (blue line) as a function time and energy The energy delivery as function of time is traced (gray line). Left side: 1064 nm Nd:YAG laser, power: 6W, CW, back and forth motion: 100 mm/s).

Using these data, it is possible to trace for a given position the maximum measured temperature and the temperature determined by mathematical modeling. For both 980 nm and 1064 nm, (Figures 5 and 6), it appears clearly that they are comparable. In Figure 5, one can observe that at the time range of 550 ~850 s, laser energy increases, but the maximum temperature obtained from simulation fluctuates. The explanation is the following:

The cannula is moved back and forth onto 100 mm in the fat layer during the laser lipolysis procedure. Consequently, for a given location (here the maximum temperature recorded during the complete treatment of the fat volume), the maximum temperature decreases. However, when the surgeon moves the cannula to a new position, heat transfer leads to an increase temperature in the vicinity of the previous position. The maximum surface temperature never exceeded 41°C.

### Total energy versus fat volume reduction

Laser irradiation induces a progressive built up of the temperature and consequently thermal damage is observed. The volume of tissue where thermal damaged (Ω ≥ 1) was considered to determine the fat volume reduction.

**Figure 7:**
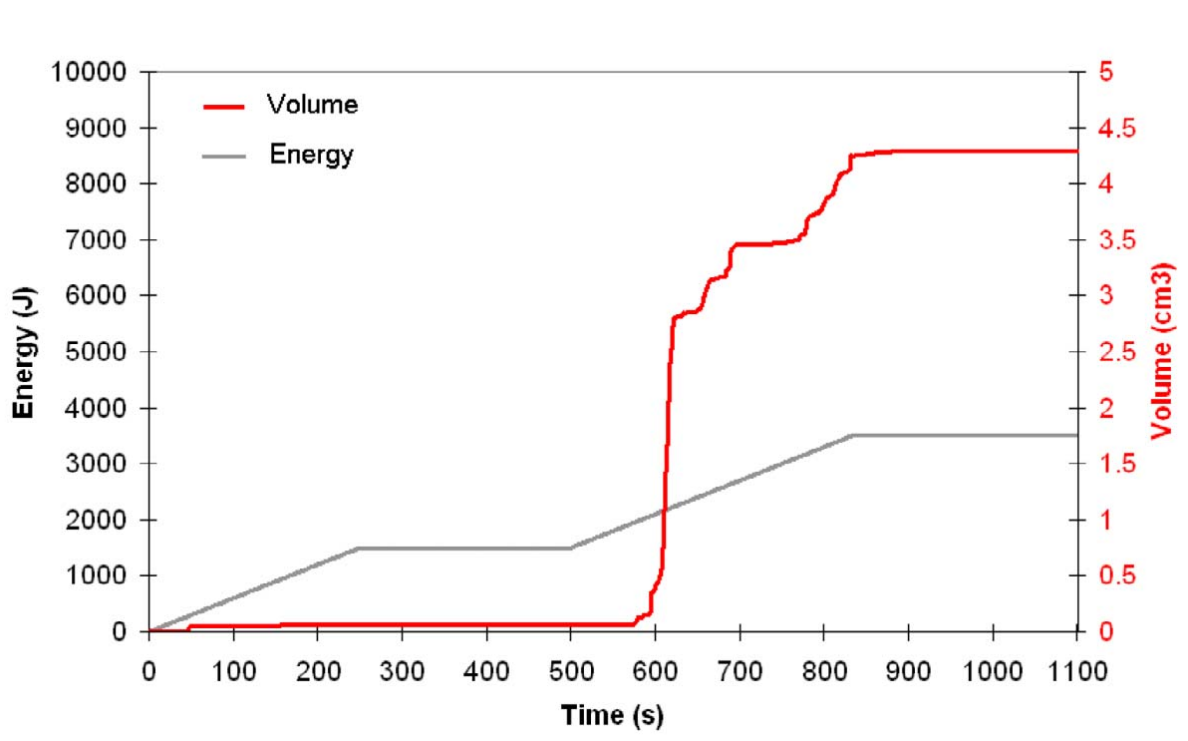
Volume reduction (red line) determined by numerical simulation as a function time and energy (980 nm-diode laser). The energy delivery as function of time is traced (gray line). Right side: 980 nm diode laser, power: 6W, CW, back and forth motion: 100 mm/s.

**Figure 8:**
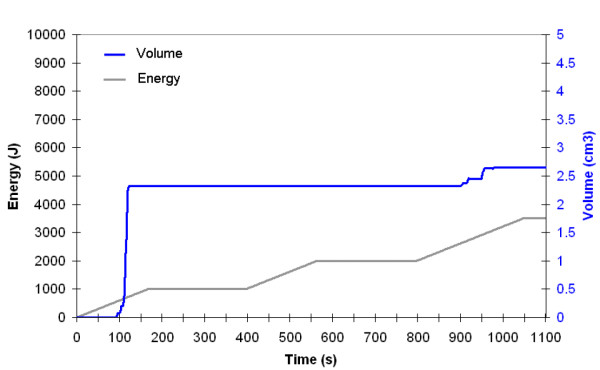
Volume reduction (blue line) determined by numerical simulation as a function time and energy. The energy delivery as function of time is traced (gray line). Left side: 1064 nm Nd:YAG laser, power: 6W, CW, back and forth motion: 100 mm/s).

At the end of the procedure, for 980 nm, a total energy of 3500 J is delivered. The volume for which Ω ≥ 1 is 4.3 cm^3^. (Figure 7) For 1064 nm, a total energy of 3100 J is delivered. The calculated volume reduction is 2.6 cm^3 ^(Figure 8). The fact that the cannula was not reciprocated exactly the same way in the left (980 nm) and the right (1064 nm) side of the submentum easily explains this difference due to a lower energy in the right side. Besides the fat volume reduction due to temperature elevation, the volume reduction due to the mechanical insertion and back and forth motion inside the fat layer must be considered. Each fiber insertion during the back and forth motion creates a mechanical damage along the fiber with a resulting channel. This channel is even produced without firing the laser. When considering the total number of channels created by the back and forth motion of a 400 *μ*m fiber, an additional 3.5 cm^3 ^must be added to the fat volume reduction induced by heating.

Consequently thermal and mechanical damages leads to a total volume reduction of 10.4 cm^3^.

**Figure 9:**
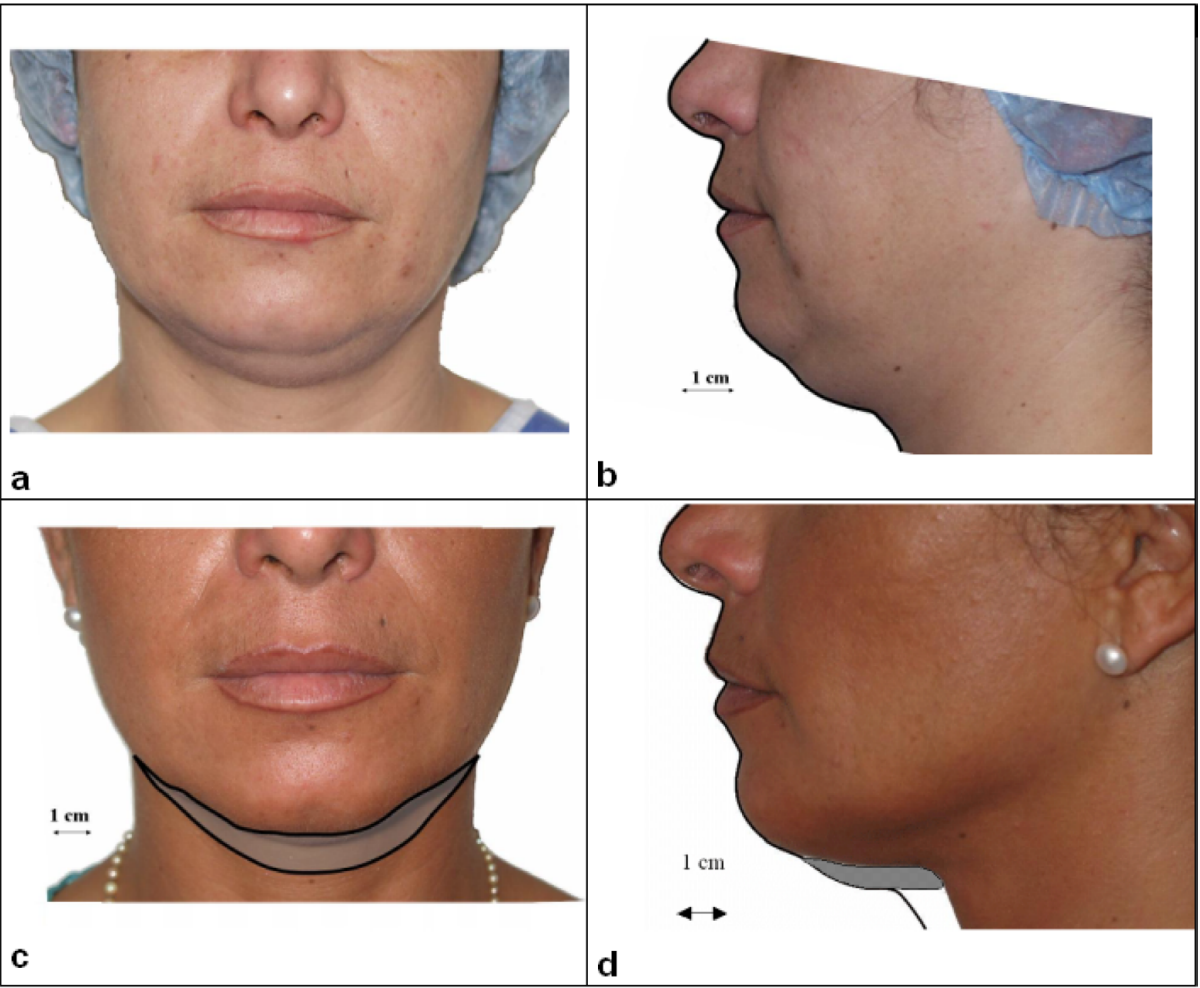
shows pictures of the patient before and at 6-month follow-up. - a and b: before laser lipolysis, - c and d: 6 months after laser lipolysis. Right side: 980 nm diode laser, power: 6W, CW, back and forth motion: 100 mm/s, total energy: 3500 J). Left side: 1064 nm Nd:YAG laser, power: 6W, CW, back and forth motion: 100 mm/s, total energy: 3100 J)

This volume was compared to the fat volume reduction obtained in this patient 6 months after laser lipolysis. Before and 6-month follow up pictures were used to quantify the fat volume reduction (Figures 9a, b, c, d). Using these calibrated images, volume reduction was calculated. A 10 cm^3 ^± 1 cm^3 ^volume reduction was obtained.

At 6 month-follow up, skin tightening was also observed. Since this mechanism was already described using non-ablative laser remodeling, numerical simulation was performed to know the temperature distribution inside the skin layer.

### Temperature distribution inside the skin layer

**Figure 10:**
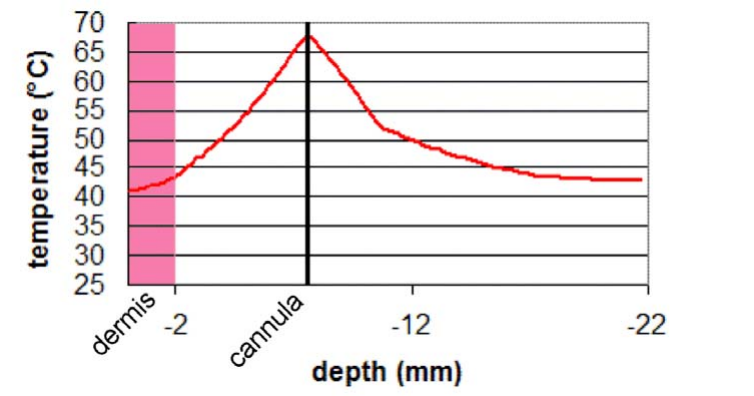
maximum temperature elevation recorded inside the skin during laser lipolysis. Parameters: 980 nm diode laser, power: 6W, CW, back and forth motion: 100 mm/s, total energy: 3500 J.

The temperature distribution inside the skin layer is displayed in Figure 10. Due to heat diffusion from the fat layer, the dermis and the epidermis are also heated. The temperatures reached inside the lower dermis (1.5 mm) and the reticular dermis (0.5 mm) are respectively 44°C and 42°C. The temperature calculated at the surface of the epidermis (41°C) is in agreement with that measured by the infrared camera.

## Discussion

Laser lipolysis is a new technique still under development. The main objectives of this technique are faster recovery, less operator effort and skin tightening [7]. The use of the 1064 nm-Nd:YAG laser and the 980-nm diode laser as an auxiliary tool has refined the traditional lipoplasty technique. Under anaesthesia, a small puncture or incision is made in the skin and the laser light is conveyed through the insertion of a micro-cannula (with a diameter of 1 mm) into which an optical fibre has been inserted. The surgeon is able to see where the end of the cannula is at all times due to a visible red light of the aiming beam that shines through the skin. The surgeon can then move the cannula back and forth and laterally to effectively and evenly liquefy the fat in the treatment area. Due to the small size of cannulas used (1 mm) this liposculpture treatment is considered to be minimally invasive when compared to traditional liposuction techniques, meaning that delicate areas such as the face, forearms, upper abdomen and knees can be treated using this method. The fat is then disposed of via the body's natural processes.

The interaction of the laser with the tissue is achieved by the absorption of the laser energy by the receptive chromophores, thus producing sufficient heat to cause the desired thermal damage. The heat acts on the fatty cell and, the extracellular matrix to produce both reversible and irreversible cellular damage. Several studies have demonstrated that for low energy settings and both 980 nm and 1064 nm, a tumefaction of the adipocytes is observed leading to an increase of their diameter up to 100 *μ*m [4,5]. The heat generated by the laser would alter the balance of sodium and potassium of the cellular membrane, allowing the free transport of extracellular liquid to the intracellular atmosphere. For higher energy settings rupture of adipocytes, coagulation of collagen fiber and small vessels are observed [3,5]. Due to the rupture of the membrane, lipases liberated by the adipocyte are responsible for the liquefaction of the tissue, which further facilitates the subsequent aspiration. Through the liquefactive effect of the laser, the back and forth movement of the cannula is performed much more easily than when performing the conventional liposuction technique. The fact that heat induces also coagulation of small vessels in the fat tissue is very important since this phenomenon facilitates the liposuction through less trauma and bleeding. Liposuction removes significant amounts of fat, serum and blood. In cases wherein large amounts of fatty tissue are to be removed, a physiologically significant loss of blood can provoke metabolic alterations. In this way, laser-assisted liposuction offers the advantage of removing larger volumes of fat without hemodynamic repercussions [4].

To date, mathematical modeling of laser lipolysis has never been proposed. This task was performed to assist in providing a better understanding of the laser lipolysis process and possibly to determine the optimal dosage as a function of the volume of fat to be removed. Our model remains a mathematical model, implying that errors may appear owing to the considerations and simplifications required to realize it. Generally, such errors appear because of inaccuracy of the optical, thermal, and damage properties that are critical points in the model's set of equations. In fact, these properties play a key role in the accuracy of the results achieved. Many methods have been presented to calculate these properties but still we see differences in the values presented by the different groups, which reflect the difficulty of measuring these properties. The problem is increased by the reliance of the properties on different variables (temperature, damage) over time. This makes the deviation neither linear nor regular [9]. Moreover, it is very difficult to standardize the surgeon's technique during laser lypolysis. For, this reason and in order to compare the results of our calculations to clinical data already reported in the literature, only submental laser lipolysis was considered. Video recordings were used to gain a better understanding of the back and forth movement of the cannula during laser lipolysis (movie 01) in order to consider them in our mathematical model. Infrared video recordings were also performed in order to compare the actual surface temperatures to our calculations.

These video recordings have provided useful information on the position of cannula during laser lipolysis and in particular how this cannula is moved back and forth inside the fat layer. The parameters gained from these video recordings are comparable to those reported in the literature. For example, Ichikawa et al inserted the cannula into the target layer of the subcutaneous fat approximately 1 cm below the skin [7]. This cannula was moved back and forth several times. The step consisted in irradiating the fat layer by using the cannula with a different angle while maintained on the same plane. Consequently, these video recordings confirm that our mathematical simulations are transposable to the surgical practice.

Before attempting to compare the parameters used for simulation to those usually reported in the literature, the following comments must be made: 1) to the best of our knowledge no previous publications have reported mathematical modeling of laser lipolysis 2) Several clinical papers were published. However in most of them, laser parameters in particular the total energy of a given volume are not reported. At last, if clinical before and after photographs are presented, the reduction of fat volume is usually not reported, except in one paper where magnetic Resonance Imaging before laser lipolysis and at 3-month follow-up was used to quantify this volume [6].

Besides the fat volume reduction due to temperature elevation, the volume reduction due to the mechanical insertion and back and forth motion inside the fat layer must be considered. Histologic studies have showed that a channel was produced due to fiber insertion. Rupture of adipocytes and fragments of adipose cell membrane were observed inside this channel [3,5].

It is interesting to compare the parameters used in our mathematical simulations to those usually reported in the literature, for the submental zone. However only a few clinical studies were published and in most of them important data are missing. Goldman, in a clinical report on submental Nd:YAG laser lipolysis on 82 patients, observed on histology, the coagulation of small blood vessel in the fatty tissue and the rupture of adipocytes [3]. Unfortunately, no data are given on the total applied energy, and the exact fat volume reduction. Prado et al used a Nd:YAG laser and applied 400 J for in the submental zone [2]. Key performed submental laser lipolysis with a 1320 nm laser using 560 to 1040 Joules. However, for these two clinical studies, the reduction of the fat volume remained unknown. In order to quantify the reduction of the fat volume, ten patients treated by Kim et al underwent an MRI pre-procedure and 3-months after 1064 nm-laser lipolysis. Looking at the specific sites, the submentum with a baseline average volume of 20 cm^3 ^had a greater reduction (25%) compared with other, larger treatment sites, suggesting a dose-response relationship [6]. In the submental zone, approximately 3000 J was applied to get a mean volume reduction of 5.2 ± 2.8 cm^3^. This is comparable to the volume reduction observed in our study: 10 cm^3 ^± 1 cm^3 ^for a total energy of 6600 J.

At last, the mechanical cannulation of body fat tissues leads also to a fat volume reduction. Our calculations demonstrate that it represents one third of the total volume.

As a result, the interaction between the laser and the biological tissue produce a reduction of the volume of fat as well as remodeling of the collagenous tissue, with clinically evident skin retraction. The heating of deep reticular dermis is confirmed by the infrared measurements and by our calculations. These temperatures are comparable to those already reported when laser irradiation was performed with a non-ablative laser [14,15]. They induce an inflammation process inside the deeper dermis. The wound repair that follows the laser treatment leads to the creation of new collagen and elastin fibers and consequently tissue tightening. This phenomenon is confirmed by Badin et al who observed after histological analysis that the collagen denaturing performed in the deep reticular dermis and the conjunctive septum of the subcutaneous tissue constituted a proinflammatory stimulant followed by vascular proliferation and collagen neosynthesis [4]. At last, the resulting skin retraction observed on the patient confirms these claims and is in accordance to previous clinical studies [16,6].

## Conclusion

Laser lipolysis can be studied by numerical simulation. Temperature elevation measured on skin surface and fat volume reduction measured after treatment are comparable to those determined by calculation. The interaction between laser and the adipocytes causes lipolysis with reduced bleeding and its effects on collagen tone promote collagen retraction and skin shrinkage. It is remarkable that by means of a few simplyfing assumptions, laser lipolysis can be described by a very tractable and intuitive model. The numerical model shows a very good fit with the experimental data. This model should serve as a useful tool to simulate and better understand the mechanism of action of laser lipolysis

## Supplementary Material

Additional file 1 video recording of a laser lipolysis procedure inside the submentum. conventional and infrared video recordings are performed simultaneously. Parameters used during the procedure were the following: left side; 1064 nm Nd:YAG laser, power: 6W, CW, back and forth motion: 100 mm/s. right side: 980 nm diode laser, power: 6W, CW, back and forth motion: 100 mm/s Click here for file

Additional file 2 video recording of a laser lipolysis procedure inside the submentum. infrared video recording (right) and movie obtained by numerical simulation using the same laser parameters (left) Parameters used during the procedure were the following: 980 nm diode laser, power: 6W, CW, back and forth motion: 100 mm/s. Mathematical modeling of surface temperature appears to be similar to surface temperature recorded during laser lipolysis of the submentum. Click here for file
